# Relationships between numerical score and free text comments in student evaluations of teaching: A sentiment topic analysis reveals the influence of gender and culture

**DOI:** 10.1371/journal.pone.0324619

**Published:** 2025-06-13

**Authors:** Fiona Kim, Xiongwen Ke, Emma L. Johnston, Yanan Fan

**Affiliations:** 1 School of Mathematics and Statistics, UNSW, Sydney, New South Wales, Australia; 2 School of Mathematics and Statistics, Central South University, Changsha, Hunan, China; 3 School of Life and Environmental Sciences, University of Sydney, Camperdown, New South Wales, Australia; 4 Data61, CSIRO, Eveleigh, New South Wales, Australia; Kitami Institute of Technology, JAPAN

## Abstract

Student evaluations of teaching (SET) have been widely used by university staff to inform decisions on hiring and promotion. In recent years, an increasing body of research has revealed that student evaluations may be systemically affected by students’ own conscious or unconscious biases. In this article, we study a data set from an Australian university, where both numerical and text survey responses were available in large quantities. Our study directly linked comments to numerical ratings, we developed approaches to convert text to quantitative data in the form of topics and sentiment scores, and make use of Bayesian ordinal regression techniques to identify drivers of SET scores. Our analysis of text identified 6 teaching dimensions that students discuss in their comments. Our findings suggest that students’ SET ratings were correlated primarily with the personal characteristics of the lecturer (such as approachability, and being nice) than measures related to teaching dimensions such as course content and assessment. We found a positive gender effect towards the majority gender in a faculty, possibly reflecting students’ gendered expectations. Finally we found that lecturers with a non-English language background were consistently rated lower by the student population, and this effect manifests strongly in local students.

## 1 Introduction

The prominence and use of student evaluation of teaching (SET) data within academic institutions has long been contentious. Several scholars have questioned whether SET can really measure teaching effectiveness [1, 2], leading to many institutions now regarding these as measures of student experience. Numerous recent studies of numerical data in SET surveys have found evidence of gender bias, men are consistently rated higher than women [3–5]. While [6] found gender bias in both directions, depending on the students’ expectations of gender roles, and [7] found no overall gender bias, but a gender affinity effect, where students evaluate a teacher of their own gender best. More recently, [8] found evidence of gendered perception of bias, when a group of students were alerted to the possibility of bias in SET ratings, the experimental data showed that female students were less likely to modify their ratings compared to male students, potentially implying that female students did not perceive themselves to be biased towards female instructors.

There is also a growing number of studies which suggest gender groups are being evaluated on different criteria. For example, men are evaluated according to the stereotypical male-dominant characteristics such as authoritativeness, intelligence and leadership abilities whereas women are assessed on their compassion, responsiveness and nurturing qualities [3, 9, 10]. [11, 12] argued that gender bias may not be easily detectable by quantitative data, and even when numerical responses do not show gender bias, text responses can show interesting gendered differences. [11] found that the words “caring, understanding, intelligent, helpful, interesting, and fair" are commonly used to describe best teachers of both genders. While men were more likely to be described as caring, understanding and funny, women were more often described as caring, helpful and kind. [13] studied qualitative data that showed male teachers received comments on subject knowledge while female teachers received comments more in terms of service to students. [14] showed that students’ comments aligned with conformity to gendered expectations (e.g. role congruity theory, [15]), and punished those who did not conform (e.g. the backlash theory, [16]). [16] also conducted experiments which suggested that perceived gender nonconformity may help to explain backlash against female professors, particularly in high-status departments.

Text responses, in addition to the numerical overall satisfaction rating can shed light on the rationale behind the ratings. While text responses are routinely collected alongside the numeric ratings, university administrators routinely ignore the accompanying text responses in higher level summaries. As a result, information in the comments have remained largely available only to individual lecturers, whilst their overall message has remained difficult to extract for university management, the correlation between these text comments and the numerical score is largely unknown. Work exploring the links between text and numerical data are scarce, partly due to the challenging nature of such data. [17] conducted a study of a large SET data set from RateMyProfessors.com in which they carried out separate analyses of the ratings and text responses, the authors found that the average ratings were lower for women professors compared to men professors. In addition, consistent with previous similar studies [12, 14], they found differences in word usages, where women professors tend to be praised for their kindness and supportiveness while men were praised for being funny and intelligent. Interestingly, their results appear to suggest men professors receive higher ratings even though the comments appear to be less positive compared with women professors. [18] used a relatively small data set from 20 engineering subjects, and sentiment analysis techniques to examine the association between the numerical score and identified words from text responses. They found for example, the word “lectures" was frequently used but occurs in both positive and negative comments. The word “content" was mentioned in negative responses, while the word “lecturers" was mentioned mostly in a positive way and associated with high numeric ratings. [19] studied a data set from engineering, where the authors pre-determined a number of subject and educator attributes, for example “learner engagement", “student support", “teaching quality", “communicative" etc., where these attributes were then matched to the text data. They found that the subject attribute the students mentioned most frequently was “teaching quality" and the most frequently mentioned educator attribute was “organised". The terms “approachable" or “engaging" were associated with a positive satisfaction score. In these analyses, no additional covariates were included.

In this article, we explore the multi-faceted relationship between students’ numerical ratings of teaching quality and their free text feedback, as well as the influence of gender and culture on these responses. We analyse data from several faculties across a large teaching and research intensive Australian university over a 7 year period. To do so, we consider a two stage approach, in the first stage, we identify topics frequently discussed in the text responses, and assign each student’s responses to these topics. We then give sentiment scores to each student comment (under each topic). These individual topic-level sentiment scores are then used to create numerical covariates suitable for statistical analysis. In the second stage, we propose a novel computational algorithm and run a Bayesian ordinal regression model incorporating a large number of covariates, using variable selection techniques to determine which relationships are statistically important.

In the rest of this paper, we first describe the SET data set we used in the analysis followed by description of our methodology for converting text into data and the statistical modelling and computational approach. Technical details of the Bayesian model and computational strategy are deferred to Supporting Information. We then provide results and some discussion of the findings of our modelling, and conclude with a summary of the main contributions from the paper.

## 2 Data

We used de-identified data from SET surveys collected over a 7 year time period (2010-2016) covering all teaching terms, from a large research and teaching intensive Australian university. Data was collected from several administrative units called faculties, such as Faculties of Science, Arts and Medicine etc. We focus on the response that students provided to the final survey question, which asks students to indicate where on the Likert scale (from a scale of 1 to 6 corresponding to strongly disagree, disagree, moderately disagree, moderately agree, agree, strongly agree) they would rate how satisfied they were with the quality of their lecturer’s teaching. Students were also asked to provide comments through a free text field, elaborating on the best features of the lecturer’s teaching.

The data set is made up of the following variables for each individual survey: Course ID, student’s cumulative weighted average marks (WAM) in a semester, total students (in the class), type of course (postgraduate, research or undergraduate), lecturer gender (male or female), lecturer English or non-English speaking background (an indicator for language and ethnic background), student gender (male or female), student culture (using the student’s residency status of local or international as a proxy for culture, SET score (on a Likert scale of 1 to 6) and best features (free text field).

The final data set comprised of 149,292 individual student surveys, collected from 3,063 unique courses and lecturers. The data was split across the five main academic faculties as follows: 32,852 in Arts/Social Science (ART); 40,145 in Business/Commerce (COM); 28,272 in Engineering (ENG); 6,630 in Medicine (MED) and 41,393 in Science (SCI). Lecturer and student composition vary across the faculties. Table 1 shows that ENG had the highest male to female staff ratio, as well as male to female student ratio. COM and SCI also had high male to female staff ratios but ART and MED had a higher female to male staff ratio. The proportion of staff with English speaking background was always higher than 50% except for COM and ENG where it was similar. Across the university, student gender was equally distributed, but within faculties, there was considerably more female students in ART and Medicine and the reverse was true for ENG and SCI. Interestingly the distribution of local and international students mirrored that of the staff.

**Table 1:** Breakdown of demographics from the SET data set by faculties and across the entire university (ALL). Across the rows are: Proportions of male and female lecturers (LM, LF), lecturers with English and Non-English background (LE, LNE), student gender, male and female (SM, SF) and student background, local or international (SL, SI).

	Lecturers		Lecturers		Students		Students	
	LM	LF	LE	LNE	SM	SF	SL	SI
ARTS/SOCIAL SCIENCE	40%	60%	71%	29%	31%	69%	75%	25%
BUSINESS/COMMERCE	66%	34%	50%	50%	51%	49%	56%	44%
ENGINEERING	82%	18%	50%	50%	75%	25%	52%	48%
MEDICINE	46%	54%	74%	26%	40%	60%	77%	23%
SCIENCE	60%	40%	66%	34%	55%	45%	75%	25%
ALL	56%	44%	62%	38%	50%	50%	66%	34%

## 3 Method

### 3.1 Conversion of text to data

Our work here focuses on the comments within the free text field based on student comments describing the best features of the lecturer, with the goal here to convert this text into numerical data, which can then be used in a secondary step for statistical inference. In doing this, we took a semi-supervised approach where a combination of machine and human input were used to first identify topic groups contained in the texts. For each survey, a sentiment score was then assigned to each of the topic/s contained in the survey response, thus creating new topic-sentiment variables from the text information.

We began by first cleaning and pre-processing the documents by tokenising the sentences using ‘ . ’ ,  ‘ , ’ , ‘!’ , ‘?’, ‘-’ as an indicator for a new sentence. Subsequently, all punctuation marks were removed from the text. These sentences were then converted to lower case and a spell-check of each word was performed using the package *autocorrect* [20] and typos of words which were missing a space were split using the package *wordninja* [21].

Since most responses were short, each survey text document contained at most a few sentences. We made the following assumptions:

- Surveys from the same course taught by the same lecturer are correlated, and the within course and lecturer dependence can be captured by a random effects model;
- Each student responds with one to several short sentences;
- Each sentence can contain multiple topics;
- Each non response expresses a sentiment value of 0.

Item one above ignores the fact that the same student can give several surveys for the same course with multiple lecturers, as well as across other courses. However, in this data set, a large number of students had only one or two surveys (not all courses carry out surveys each time it is taught), and 85% of the students had 5 or less surveys. We therefore ignore the modelling of student effect. Item four above deals with missing responses, i.e., when the student chose not to provide any comments in some or all of the topics. We standardized the sentiment scores to vary between -1 and 1, with sentiments above 0 being positive and less than 0 being negative. The majority of the sentiments for those who provided comments were positive, since the question asked the students to state what they considered to be best features. However, some sentiments were negative. Therefore we set those who did not offer a comment as 0, not having anything particularly positive or negative to say about the best features.

Table 2 shows the steps we used to convert text to quantitative data. We began simplifying text by considering all *noun phrases* in the documents, since in short sentences, the noun phrases provided a good indication for the topic of the discussion, and can seed the list of keywords associated with distinct topics. In Step 1, using the *textblob* [22] package we were able to extract all noun phrases from the comments, which provided us with more context to perform the classification. We then produced a frequency count of all noun phrases identified in Step 2.

**Table 2:** Steps in the transformation of text to numerical data using nouns and noun phrases to determine topics, which are then assigned a sentiment value.

Step 1	Keyword Tagging: Identify all noun phrases from the comments;
**Step 2**	**Keyword Count:** Produce the frequency counts for the list of noun phrases;
**Step 3**	**Topic Determination:** In conjunction with subject matter experts;
**Step 4**	**Seed lists:** Produce a list of seed words for each topic based on frequency

To determine topics in Step 3, we combined prior expert knowledge from multiple stakeholders in the higher education domain (including professors, students and the Associate Dean of Education) together with prior findings of common topics found within the Australia higher education setting. The base topics (Learner Engagement, Learning Resources, Skills Development, Student Support, Teaching Quality) were reported from a large scale analysis of Student Experience Surveys conducted on behalf of the Australian Government Department of Education and Training [23].

To determine the relevance of the topics defined by the Social Research Centre, we took the top 200 available frequency counts obtained in Step 2 and then all stakeholders worked independently and then collaboratively to manually cluster the noun phrases into the most appropriate groups based on similarities and the topic framework set out. The end result included some new topics derived from splitting existing topics (if there were too many noun phrases) and topics were also merged or removed entirely (if there were too few noun phrases associated with the topic).

**Fig 1:**
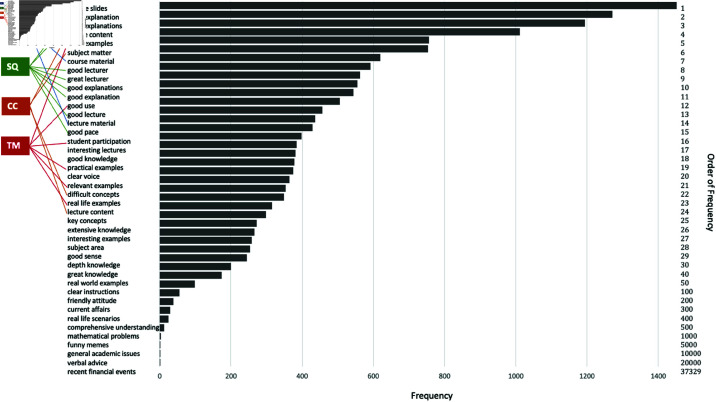
Illustrative diagram of the top noun phrase frequency and topic assignment process for our data set. *x*-axis shows the frequency of appearance of the noun phrases listed on the left axis, while the rank of their frequency is indicated on the right axis. Topic membership is indicated by color. In this illustration the topics are teaching and learning resources (TL), staff quality (SQ), course content (CC) and teaching methods (TM).

The choice of the top 200 noun phrases to use for topic determination was due to the fact that the frequencies of the noun phrases significantly decreased beyond this point. As can be seen in Fig 1, the frequency of noun phrases have dropped from order of thousands to below 50 beyond the top 200. An illustrative diagram of the noun phrase to topic allocation is given in Fig 1.

In Step 4, once the topics have been defined using the top 200 noun phrases, we identified additional singular words within this list of noun phrases that can inform the topics and combined this to expand the original seed list. Then, the seed list was expanded for each topic by assigning the remaining unclassified noun phrases, provided the noun phrase was a high match with one of the existing seed list words in a topic by a similarity score of 0.85 or more. This similarity matching was based on the cosine similarity using Google’s universal sentence encoder which contains pre-trained sentence level embeddings [24]. Google’s universal sentence encoder was designed to be general purpose and has been proven to perform well. To assess performance, we found that a random sample of 100 noun phrases showed the 0.85 cut-off produced an accuracy of 95% relative to manual classification.

In Step 5, sentences that contained any word or noun phrases from the seed list were then allocated to that corresponding topic. If a sentence contained multiple matches then it would be assigned to those multiple topics. If there was no match for a sentence with the seed list of words or noun phrases, then the sentence was assigned to the miscellaneous category which accounted for 19.7% of all comments. A discussion of contents of the miscellaneous category is given later in Sect 4.1.

In Step 6, with all sentences classified into one or more of the topics, the text in its raw form (i.e., with the original punctuation) was used to determine the sentiment, allowing us to account for the magnitude of the sentiment as emphasised through the use of capitalisation, punctuation marks and emoticon symbols.

From each sentence, a sentiment score was assigned using python’s *VADER* package [25] which incorporates a sentiment lexicon that was developed for microblog-like contexts. The sentiment lexicon behind VADER was designed to account for both polarity and intensity expressed in social media contexts and is normally applicable to sentiment analysis in other domains. This is well-suited for our purpose as student comments tend to be short, concise and are informal. Just over 7,500 lexical features are contained in the package with validated valence scores that indicate both the sentiment polarity (negative/positive), and the sentiment intensity (–4 to +4). For example, the word “okay” has a positive valence of 0.9, “good” is 1.9, and “great” is 3.1, whereas “horrible” is –2.5, and “sucks” (or its slang “sux”) are both –1.5. The lexicon also features a full list of Western-style emoticon (e.g., :-) and :( ) and sentiment-related acronyms and initials (e.g., LOL and WTF). The compound score was computed by summing the valence scores of each word in the lexicon, adjusted according to the rules, and then normalized to be between -1 and +1.

Once a sentence has been assigned to a topic and had its corresponding sentiment score calculated, the scores for all sentences belonging to the same comment and topic were averaged. The resultant feature for our model was then a sentiment score for each respective topic that the student had a comment on, and each comment can fall under multiple topics. While students who did not provide any written feedback (in any of the topics) were assigned a neutral sentiment score of 0.

Once we had categorised the free text to topics, and each sentence had a sentiment score value between -1 and +1, we could now analyse the text information via sentiment scores. For each survey response we created a set of new variables, corresponding to the sentiment scores of that survey for each topic. These new variables were used to study how each topic was related to the numerical ratings (on the Likert scale), together with other covariates such as gender and/or cultural backgrounds of lecturer and students.

### 3.2 Bayesian ordinal regression modelling

The cumulative logistic model [26] is perhaps the most commonly used model linking the ordered categorical response variable to a set of covariates. The model with random effects is defined as follows. Let the vector of ordinal SET score response be represented by $Y=(y_{1},...,y_{n}{)}^{\prime }$, where $y_{i}=1,\ldots ,6$, and set a vector of latent variable $Z=(z_{1},...,z_{n}{)}^{\prime }$ where the *i*th latent variable *z*_*i*_ is given by

$$z_{i}\sim logistic(X_{i}\beta +T_{i}b,1).$$ (1)

$X_{i}$ is the *i*th entry of an n×p covariate matrix corresponding to the *p* fixed effects such as lecturer gender, student gender, WAM and $T_{i}$ is the *i*th entry of an n×q covariate matrix taking value of 1 if the survey belongs to the *i*th course and lecturer combination, and 0 otherwise. b is the corresponding vector of random effects parameters.

The latent variable *z*_*i*_ is related to the observed ordinal response *y*_*i*_ via


$$y_{i}=\left\{ \begin{array}{cc} 1, & \text{ if }z_{i}\leq \gamma_{1}\\ s, & \text{ if }\gamma_{s-1}<z_{i}\leq \gamma_{s},\;\text{ for }s=2,\ldots ,S-1\\ S, & \text{ if }z_{i}>\gamma_{S-1} \end{array}\right.$$

where *S* = 6 is the number of ordinal response categories, and $\gamma =(\gamma_{1},...,\gamma_{S-1})$ are unknown cutoff points.

The cumulative probabilities takes the form


$$P(y_{i}\leq s)=P(z_{i}\leq \gamma_{s}|X,T)=\frac{\exp(\gamma_{s}-X_{i}\beta -T_{i}b)}{1+\exp(\gamma_{s}-X_{i}\beta -T_{i}b)}$$

and the cumulative logit model is of the form

$$\log\left(\frac{p(y_{i}\leq s|X,T)}{p(y_{i}>s|X,T)}\right)=\gamma_{s}-X_{i}\beta -T_{i}b,\;s=1,\ldots ,S-1$$ (2)

where Eq 2 provides a log-odds interpretations for the fixed parameters β, see [5].

### 3.3 Prior specification

For problems where there’s a large number of fixed effect parameters, we may wish to use a prior that allows us to decide how strongly we would like to shrink some of the coefficient parameters towards 0, so as to tease out the most statistically significant effects. A number of variable selection priors can be used [27, 28]. Here, we consider the Bridge prior [29–31], which offers some flexibility over alternatives and allows us to develop an efficient computational algorithm based on the partially collapsed Gibbs sampler of [32], increasing computational speed by several orders of magnitude.

For the fixed effect parameters β, we consider the Bridge prior of the form

$$\pi (\beta |\lambda ,\alpha )=\prod_{j=1}^{p}\frac{\lambda^{1/\alpha }}{2\Gamma (1+1/\alpha )}\exp\left(-\lambda {\left|\beta_{j}\right|}^{\alpha }\right),\;0<\alpha <1.$$ (3)

Smaller values of α will give stronger shrinkage towards 0. Here we follow [31] and set $\alpha =\frac{1}{2}$, we call this the $L_{\frac{1}{2}}$ prior. An additional level of control over the amount of shrinkage towards zero can be done via the hyperprior for λ. However in this article, we simply set a fixed value for λ so as not to put too much additional prior shrinkage on the βs.

For the prior of the cutoff points $\gamma =(\gamma_{1},...,\gamma_{S-1})$, we have that for s=1,…,S, $p_{s}=P(\gamma_{s-1}<z<\gamma_{s})=F(\gamma_{s})-F(\gamma_{s-1})$, and $\sum_{s=1}^{S}p_{s}=1$, so that


$$\gamma_{1}=F^{-1}\left(p_{1}\right),\;\gamma_{2}=F^{-1}\left(p_{1}+p_{2}\right)\;\ldots ,\gamma_{S-1}=F^{-1}\left(p_{1}+p_{2}+\cdots +p_{S-1}\right),$$

where *F* the CDF of the logistic distribution. We can assign a symmetric Dirichlet prior to $\left(p_{1},p_{2},...,p_{S}\right)$, with concentration parameter *a*>0, and by change of variable from the equation above, we have


$$\pi (\gamma_{1},\gamma_{2},...,\gamma_{S-1}|a,v)=\frac{\Gamma (aS)}{\Gamma (a{)}^{S}}\prod_{s=1}^{S}[F(\gamma_{s})-F(\gamma_{s-1}){]}^{a-1}\prod_{s=1}^{S-1}f(\gamma_{s})$$

where f(·) is the density of F(·).

Finally for the random effects b, we set the prior for $b\sim N(0,\Lambda^{-1})$ with unknown precision matrix $\Lambda^{-1}$. We assign a conjugate prior to Λ such that


$$\Lambda \sim Wishart(\delta ,P^{-1})$$

where $P^{-1}$ is the precision matrix. We used *a* = 1.1, δ=q, and the identity matrix for P throughout the analysis. The model is largely insensitive to the choices of *a*, δ and P.

We adopt a Bayesian Markov chain Monte Carlo (MCMC) approach for posterior computation, extending the partially collapsed Gibbs sampler of [31] to the ordinal regression setting. Details can be found in Supporting Information.

### 3.4 Consensus Monte Carlo

When the sample size of the data is large, it can be helpful to be able to run parallel computations to reduce computational burden. [33] describes a simple method to compute approximate posterior distributions for big data using distributed computing, they call this consensus Monte Carlo (CMC). Denoting the full set of data by Y and *Y*_*r*_ a subset of Y (or shard *r*), and let θ denote the set of model parameters, suppose the posterior distribution can be written as


$$\pi (\theta |Y)=\pi (Y|\theta )\pi (\theta )\propto \prod_{r=1}^{R}\pi (Y_{r}|\theta )\pi (\theta {)}^{1/R},$$

where *R* is the total number of shards and the prior π(θ) is diluted depending on the number of pieces.

Then each of the *R* pieces can be computed in parallel using MCMC (or any appropriate method of choice), and if each of the pieces are sufficiently large, it is then reasonable to assume that the posteriors of each piece are approximately normally distributed, leading to an approximately normally distributed full posterior. More concretely, suppose each subset generates draws $\theta_{ri}$, i=1,…,N, the consensus posterior draws $\theta_{i}$ is given as


$$\theta_{i}=\sum_{r=1}^{R}w_{r}\theta_{ri}/\sum_{r=1}^{R}w_{r}.$$

When the Gaussian approximation is used, the weights $w_{r}=\Sigma_{r}^{-1}$ are optimal and can be estimated using the sample covariance matrix from each shard. If only a subset of θ is of interest, for example the fixed effects parameters only, then the weighted samples are obtained marginally only for the parameters of interest.

For our current analyses, the full data set can be naturally broken by the administrative unit of faculty. Carrying out the analyses separately on the smaller units at the faculty and then considering them as a whole showed that SET results can vary significantly between faculties and that results aggregated at the university level could mask important effects at the faculty level. So the CMC approach is desirable in this setting as it allows us to simultaneously understand both faculty and university level results, as well as resolving the issue around computational burden.

To set the prior, we need to ensure that the prior induced by CMC is comparable to the faculty level priors. To achieve this, instead of taking powers of the prior as in [33], we change the value of the hyperparameter parameter λ (Eq 3) to control for how much shrinkage is used. We set λ=5 for each piece, when these are combined via CMC, the induced equivalent prior is approximately the same as using λ=15 for the full model, under this setting, λ is not adding too much additional shrinkage.

All computations were carried out on a compute node of a computational cluster, Katana [34]. Running all pieces of shards in parallel, with no communications during simulations. As a comparison, running the largest shard piece took approximately 0.392 seconds per iteration, compared with 8.672 seconds per iteration when running the full data set.

## Ethics declarations

- Ethics approval: This research was approved by the UNSW Human Research Ethics Advisory Panel (HREAP), HC17088, as a negligible risk project. Consent was not required.
- Consent for publication: Publication of the work is supported by the authors and in accordance to the ethics guidelines set.

## 4 Results

### 4.1 What do students comment on?

We identified six theme groups (or topics) which are related to different dimensions of teaching and course quality: assessment (AS); course content (CC); learning environment (LE); staff quality (SQ); teaching and learning resources (TL) and teaching methods (TM) and an additional group collecting all miscellaneous topics (MS). Table 3 shows some examples of noun phrases allocated to the topics. The topic on *assessment* included comments on feedback, exams and other types of assessment tasks. The topic on *course content* included anything that refers to the content of the course, e.g., the subject matter, concepts and structure of the course, the topics in the course etc. The *learning environment* topic referred to lecture theatres and the learning experience. The *staff quality* topic included many more noun phrases, here the noun phrases described the lecturer directly, e.g., good pace, depth knowledge, approachability, clear teaching and nice guy etc. The topic *teaching and learning resources* referred to provision of slides, and lecture materials, or videos and visual aids, this is different to *course content*, which is related to the subject matter of the course. Finally *teaching method* covered techniques used for teaching, such as encouraging student participation, or the use of real life examples, and guest speakers.

**Table 3:** Representative noun phrases corresponding to the identified topics: assessment (AS), course content (CC), learning environment (LE), staff quality (SQ), teaching and learning resources (TL) and teaching methods (TM).

AS	good feedback, assessment task, final exam, helpful feedbacks. ...
**CC**	course content, subject matter, difficult concept, interesting topic, clear structure, ...
**LE**	lecture theatre, learning process, learning experience, ...
**SQ**	clear explanation, good lecture, good pace, depth knowledge, approachability, clear teaching, nice guy, good preparation, ...
**TL**	lecture slide, course material, video clip , point slides, visual aid, practice questions, ...
**TM**	student participation, real life example, case study, encourages student, guest speaker, ...

In order to assess the reliability of our topic assignment procedure, we performed validation by manually checking the accuracy of the topic assignment. To do this, we randomly sampled 200 sentences which corresponded to 245 topic assignments, and computed the accuracy, precision and recall proportions, shown in Table 4. Here accuracy (%) is defined as (TP+TN)/(TP+TN+FP+FN), where TP denotes the true positive rate (a correct topic assignment is made), TN denotes the true negative rate (a no match with the topics is correctly predicted) and FN denotes the false negative rate (a no match was found when a topic assignment was available). Precision (%) is defined as TP/(TP+FP) and recall (%) is defined as TP/(TP+FN). The breakdown of the results in Table 4 shows an overall accuracy of around 64% which ranged 48% to 82% between topics; a precision of 84%, which ranged 79% to 91% between topics and a 69% overll recall, which ranged from 55% to 91% between topics. The higher precision and recall rates compared to accuracy was due to a more conservative threshold selection to minimise false positives.

**Table 4:** Accuracy, precision and recall by topic and overall, based on manual validation of 200 randomly selected sentences. LE is omitted due to lack of samples.

Topic	Accuracy (%)	Precision (%)	Recall (%)
AS	56	83	63
CC	81	88	91
SQ	48	79	55
TL	69	91	74
TM	82	91	89
Total	64	84	69

Below are some examples of the comments assigned to the various topics, with the seed list word or noun phrase used to classify the sentence presented in **bold** and corresponding sentiment score presented in the square brackets.For example, the following sentences were correctly assigned to staff quality (SQ):

“good lecturing **style** and **pace** [0.44]"

“ **good explanation** of complex ideas excellent **communication** with students" [0.77]

“i loved his teaching **style** as it kept me interested and he made maths fun" [0.87]

Examples of course content (CC) included:

“clear **concepts** of the **subject**" [0.38]

“the **contents** are very clear" [0.44]

“which makes learning the **content** seem more practical and interesting" [0.45]

Examples of the other topics were:

AS: “quick turnaround with **assessments**" [0.00]

TL: “enough information and **materials**" [0.00]

TM: “i feel he tried to make the course relevant to us and i enjoyed coming to his **lectures**" [0.51]

From inspection of the unclassified sentences which form part of MS, there were varied reasons as to why the sentences below were left unclassified. In Example 1-3 below, the object of the sentence were not present in the text and thus it is unclear which aspect of the teaching or course was done well and hence the sentence can’t be matched to a topic. There were also sentences, such as Example 4 below, which were very niche or specific to a course terminology or random and thus genuinely don’t belong in any of the existing topics.

Example 1: “was definitely a good thing" [0.68]

Example 2: “and ability to break down information quickly" [0.31]

Example 3: “which makes the class interesting to learn" [0.40]

Example 4: “whether they be photographs" [0.00]

### 4.2 What influences SET ratings?

To fit the ordinal regression models, all non-binary covariates (including the sentiment scores) were standardised by subtracting the mean and divided by 2 standard deviations to allow for easy interpretation of results. This approach allows comparable interpretation of these covariates to the binary variables in the same model, while subtracting the mean allows for the main effects of the interactions to be more easily interpreted [35].

We fitted several models. First we used the SET score as the response variable, and fitted the cumulative logistic model using all the covariate information described in [sec:dna]Sect 2, including the sentiment values of each of the topics derived above, and interactions of lecturer, student and topic measurements. This resulted in a total of 84 covariates (see S1 Table, S1 File). Random effects accounting for course and lecturer effect are also included. We ran the PCG sampler described in the SI using 20,000 iterations discarding the initial 10,000 as burn-in. Each shard, corresponding to the five faculties, were run separately using the prior conditions set above and combined using the CMC approach.

To further understand the sentiments, we also considered those topics which were statistically significant predictors of the ratings. To do this, we convert the sentiment scores to three levels: positive, neutral and negative. Sentiment scores >0 and <0 were set to positive and negative respectively, and the remainder set to neutral (including non-responses). We then fit a separate ordinal regression model with sentiments as the response, and the same fixed and random effects variables as in the ratings model, excluding the remaining topics. Parameter estimates from these models are provided in S2–S4 Tables (S1 File). Throughout the paper, we will use the term significant to mean statistically significant at the 95% level.

### Topic relevance

Of the seven topics we derived, only three were significantly (and positively) correlated with the ratings (see S1 Table, S1 File): staff quality, teaching methods and the miscellaneous category. While students did comment on course content, assessments, teaching and learning resources, and learning environment, these were not significantly correlated with the ratings. The most dominant topic is staff quality, which refered to comments on pace of delivery, depth of lecturer’s knowledge, approachability and all other personal aspects of the lecturer, this was positively correlated across all the faculties. The remaining two topics were teaching methods and miscellaneous. The former incorporated comments related to techniques used for teaching, such as encouraging student participation, or the use of real life examples. The latter comprised of all comments that we could not classify to one of the teaching dimensions. These results suggested that ratings were primarily driven by the personal characteristics of the lecturers, and less so by teaching dimensions of interest, even though students praised these teaching dimensions in the comments.

### Gender effects

We found significant gender effects on the ratings, alongside topic sentiments. This is consistent with the topic findings that suggest personal attributes of the lecturer wereinfluencing the ratings. There were several important findings in terms of gender effects that we discuss below.

**Fig 2:**
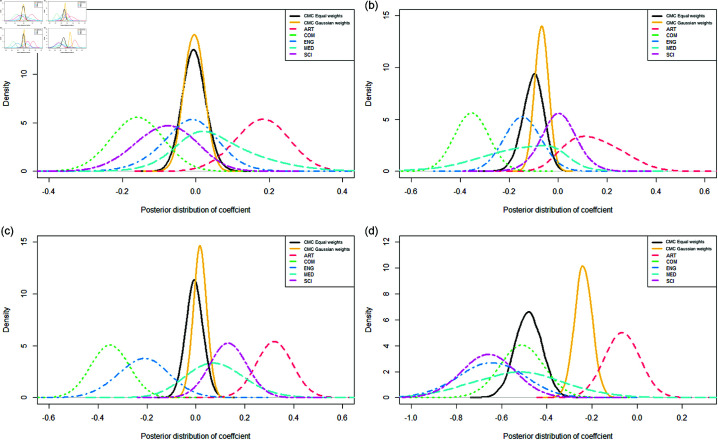
Posterior densities using CMC results with equal (black) and Gaussian (yellow) weights for the effect of being (a) female lecturer, (b) lecturer from a non-English speaking background, (c) local student rating female lecturer and (d) local student rating female lecturer from a non-English speaking background. Also plotted are the posterior densities for each faculty (Arts/Social Sciences (ART), Business/Commerce (COM), Engineering (ENG), Medicine (MED), Science (SCI)) estimated separately. The plots show how estimates can vary drastically between the faculties (i.e., positive or negative effects), compared to the combined results from the entire university, where strong individual effects may have canceled out after aggregation.

First, our results showed that the effect of gender can be positive or negative for both male and female lecturers, meaning that in some faculties female lecturers were rated higher than male lecturers and vice versa in other faculties. This had resulted in no significant difference in gender when data were pooled across the university. What we observe here is an instance of the well-known Simpson’s paradox, and information from faculty-level analysis can help us better understand the university level analyses. Fig 2a shows how the posterior distributions vary across the faculties. We see that the effects of being a female lecturer ranged from being significantly positive in Arts/Social Science (ART) (around 0.2 and the majority of posterior mass away from 0), to significantly negative in Business/Commerce(COM) (around -0.15, and again posterior mass concentrated outside of 0), while the remaining faculties fall somewhere in-between. However, we can see that when the data is analysed at the university level, as the posterior mass is concentrated on 0, meaning that there is no significant gender effect when pooling data across the university.

Second, positive effects were found in ART and MED for females lecturers (significant for ART), while negative effects were found for COM, ENG and SCI (significant in COM). An examination of the gender breakdown in our data set in Table 1 shows that ART and MED were the only two faculties where there were more females than males, in both lecturer and student numbers. The reverse is true in the remaining faculties where the gender effect is reversed. Our results give support to [6], who recently argued that gender bias occurs from students’ expectation of gender roles, and hence students in faculties with more female lecturers favor the female lecturers and the reverse is true for faculties dominated by male lecturers. The mirroring of student gender composition is interesting, suggesting that gender role models may influence a student’s choice of subject to study [36].

Third, when looking only at factors driving the topics, we see that being female was positively correlated with a higher staff quality sentiment score (S2 Table, S1 File), and the effects were significant in ART and at the university level. In other words, being female did not negatively influence the sentiments on staff quality even when they were negatively influencing the ratings (e.g. COM). Similar for teaching methods, a significantly positive effect was found for being female at the university level (S3 Table, S1 File). These results suggest that while students may be positive about female lecturers in their comments, though they may rate female instructors lower than male lecturers.

We also note the effect of gender was negative and significant for SCI and across the university for the miscellaneous topic (S4 Table, S1 File). The negative result for females in the miscellaneous topic is significant in that females appear to be more likely to be commented negatively on a range of points not closely related to our teaching topics.

Finally, we found no significant gender and topic interactions, but some significant lecturer gender and student characteristic level interactions. We found that local students were significantly more negative towards female lecturers in COM and ENG but positive in SCI and ART. Fig 2a and 2c shows the corresponding posterior distributions which again lead to a non-significant difference at the university level.

### Cultural effects

**Fig 3:**
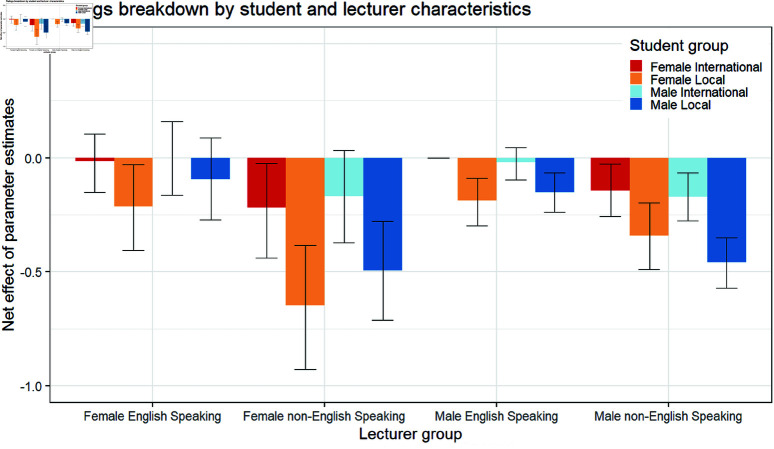
Results from the ratings model. Bars indicate the posterior mean of being in the Lecturer Female and Male with English and non-English speaking backgrounds as evaluated by local and international male and female students. The whiskers indicate 95% confidence intervals.

We found the effect of having an English speaking background to be significantly negative in COM and ENG and consistent with the overall university result of a significantly negative effect. Fig 2b shows the posteriors of the effect of being of non-English background across the faculties. Fig 3 shows that local students were particularly harsh on lecturers with a non-English speaking background, suggesting a cultural effect might be at play. Unlike the gender effect term, the effect of language background on sentiment scores remained consistent as those in the ratings model, and negative across the three topics.

## 5 Conclusion

In this article, we have analysed data from student evaluations of teaching surveys in the presence of both text and numerical information. We used a two-stage procedure where we converted the text data to topics and sentiment scores, and then applied a Bayesian ordinal regression model using the combined data set of covariate measurements and topic-sentiment scores. Unlike previous approaches where either word associations with ratings were considered [19]; or topics were analysed separately to ratings [17], our approach allowed us to make direct statistical inference about the significance of associations between topics derived from student comments and its corresponding ratings.

We used Bayesian ordinal regression with a variable selection prior to allow for potentially large amounts of covariate information to be included in the model. We developed an efficient partially collapsed Gibbs sampler, using the $L_{\frac{1}{2}}$ variable selection prior. We also showed how consensus Monte Carlo is useful in this setting, allowing us not only to make large scale computation possible, but also allowing us to simultaneously consider more detailed information from subsets of the data. The latter led us to the finding that when these data were analysed at the aggregated level (university level), any statistically significant gendered effects disappeared, illustrating an instance of the Simpson’s paradox, where the negative and positive effects cancel out when data was aggregated. This is a new and important finding because university administration should take this phenomenon into account when aggregating SET results to ensure faculties/departments do not overlook potential.

Our analyses provided two major findings: firstly, we identified six primary teaching dimensions that the students regularly mention in their text responses. While the topics are broadly consistent with previous findings [17, 23], we found that only two of these topics, staff quality and teaching methods, were correlated with the numerical ratings. A third topic, miscellaneous, which included comments not strongly related to the teaching dimensions identified, was also found to be significantly correlated with ratings. These findings provide statistical evidence to support previous studies that cast doubt on the nature of what SETs were measuring. [37] found that SETs were more sensitive to gender biases than an objective measure of teaching effectiveness (such as student performance), while [14] used qualitative methods to argue that SETs appear to be measuring gender conformity. Our findings however, suggest that among other things, SETs measure an individual lecturer’s ability to provide “clear explanation”, good “pace” of delivery, their “approachability”, as well as ability to encourage students and incorporate real life examples and motivations. Several other teaching dimensions, such as assessments and feedback, course content and lecture environments were found to be frequently discussed by the students, but they do not in fact correlate significantly with ratings. Noting that the non-significant topics were those with less emphasis on personal aspects of the lecturer, suggesting that students perceive the ratings as personal. Finally, similar to previous findings, we also found SET to be measuring several other factors, both as evidenced by the significant nature of the miscellaneous category, where students discussed a wide range of topics, as well as the significance of both lecturer and student gender and cultural backgrounds.

Our second main finding, is that even after teaching dimensions have been accounted for, there were still significant effects attributable to the lecturer’s gender, however, this effect was not the same between different faculties: positive for female lecturers in ART and MED and negative in COM, ENG and SCI. It is unclear what drives this disparity, one theory is that the positive effects may have been due to the higher female to male ratio in both the staff and student populations, and the role of gender-based expectations [6, 36]. Similarly, a significant and negative effect was attributable to staff cultural backgrounds, without the disparities observed under gender. An examination of the demographic split across faculties revealed that staff with non-English speaking background remain in minority across all faculties. In both gender and cultural terms, we observed what can be called the minority effect, that is, staff belonging to the minority group in that faculty were penalised in their SET ratings. While the effects of demographic factors were significant, unlike the findings in [37], they were on similar magnitude to the teaching dimension effects. Finally, we found that while overall ratings were negatively associated with female lecturers in some faculties, they were positively correlated to sentiment scores for teaching dimension topics, this finding echoes that of [17] using data from RateMyProfessors.com. However, female lecturers were negatively correlated with the sentiment scores for the miscellaneous topics, this was more pronounced in the faculties where female lectures received lower ratings. These findings again suggest that women may be held to higher standards than men.

One limitation of this study is that we were not able to use the text comments where students were asked to discuss how the lecturers could improve. This set of data included language that can often involve a double negative, e.g., “nothing" for improvement, which resulted in inaccurate sentiment scores. Incorporating this set of information may further enhance our understanding of the data but is beyond the scope of this paper as new methods for converting such comments to sentiment scores are required.

## Supporting information

S1 File Supplementary material. (PDF)

## References

[1] Stark PB, Freishtat R. An evaluation of course evaluations. ScienceOpen Res. 2014. doi: 10.14293/s2199-1006.1.sor-edu.aofrqa.v1

[2] Hornstein HA. Student evaluations of teaching are an inadequate assessment tool for evaluating faculty performance. Cogent Educ. 2017;6(1).

[3] Boring A. Gender biases in student evaluations of teaching. J Publ Econ. 2017;145:27–41.

[4] MacNell L, Driscoll A, Hunt AN. What’s in a name: exposing gender bias in student ratings of teaching. Innov High Educ. 2015;40(4):291–303.

[5] Fan Y, Shepherd LJ, Slavich E, Waters D, Stone M, Abel R, et al. Gender and cultural bias in student evaluations: why representation matters. PLoS One. 2019;14(2):e0209749. doi: 10.1371/journal.pone.0209749 30759093 PMC6373838

[6] Aragón OR, Pietri ES, Powell BA. Gender bias in teaching evaluations: the causal role of department gender composition. Proc Natl Acad Sci U S A. 2023;120(4):e2118466120. doi: 10.1073/pnas.2118466120 36649402 PMC9942858

[7] Binderkrantz AS, Bisgaard M. A gender affinity effect: the role of gender in teaching evaluations at a Danish university. High Educ. 2023;87(3):591–610. doi: 10.1007/s10734-023-01025-9

[8] Kim F, Williams LA, Johnston EL, Fan Y. Bias intervention messaging in student evaluations of teaching: the role of gendered perceptions of bias. Heliyon. 2024;10(17):e37140. doi: 10.1016/j.heliyon.2024.e37140 39296176 PMC11409104

[9] Bennett SK. Student perceptions of and expectations for male and female instructors: evidence relating to the question of gender bias in teaching evaluation. J Educ Psychol. 1982;74(2):170–9.

[10] Hoorens V, Dekkers G, Deschrijver E. Gender bias in student evaluations of teaching: students’ self-affirmation reduces the bias by lowering evaluations of male professors. Sex Roles. 2020;84(1–2):34–48. doi: 10.1007/s11199-020-01148-8

[11] Sprague J, Massoni K. Student evaluations and gendered expectations: what we can’t count can hurt us. Sex Roles. 2005;53(11–12):779–93. doi: 10.1007/s11199-005-8292-4

[12] Gelber K, Brennan K, Duriesmith D, Fenton E. Gendered mundanities: gender bias in student evaluations of teaching in political science. Austral J Polit Sci. 2022;57(2):199–220. doi: 10.1080/10361146.2022.2043241

[13] Sigurdardottir MS, Rafnsdottir GL, Jónsdóttir AH, Kristofersson DM. Student evaluation of teaching: gender bias in a country at the forefront of gender equality. High Educ Res Develop. 2022;42(4):954–67. doi: 10.1080/07294360.2022.2087604

[14] Adams S, Bekker S, Fan Y, Gordon T, Shepherd LJ, Slavich E, et al. Gender bias in student evaluations of teaching: ‘punish[ing] those who fail to do their gender right’. High Educ. 2021;83(4):787–807. doi: 10.1007/s10734-021-00704-9

[15] Eagly AH, Karau SJ. Role congruity theory of prejudice toward female leaders. Psychol Rev. 2002;109(3):573–98. doi: 10.1037/0033-295x.109.3.573 12088246

[16] Fisher AN, Stinson DA, Kalajdzic A. Unpacking backlash: individual and contextual moderators of bias against female professors. Basic Appl Soc Psychol. 2019;41(5):305–25. doi: 10.1080/01973533.2019.1652178

[17] Zheng X, Vastrad S, He J, Ni C. Contextualizing gender disparities in online teaching evaluations for professors. PLoS One. 2023;18(3):e0282704. doi: 10.1371/journal.pone.0282704 36928194 PMC10019737

[18] Cunningham-Nelson S, Baktashmotlagh M, Boles W. Linking numerical scores with sentiment analysis of students’ teaching and subject evaluation surveys: pointers to teaching enhancements. In: Proceedings of the Research in Engineering Education Symposium and Australasian Association for Engineering Education Annual Conference, AAEE2016, Coffs Harbour, Australia. 2016. p. 1–8.

[19] Cunningham-Nelson S, Dart S. What do students care about?: An analysis of topics impacting student evaluation survey results in engineering. In: Proceedings of the Research in Engineering Education Symposium and Australasian Association for Engineering Education Annual Conference AAEE2021. 2021. p. 1–8.

[20] McCallum J, Sondej F. Autocorrect python package. 2021.

[21] Negm MS, Mandour WS. Words of peace in the speeches of the Egyptian President, Abdulfattah El-Sisi. Corpus-Based Study. 2020;14(1):6.

[22] Loria S. Textblob Documentation. 2. 2018.

[23] Social Research Centre. Student Experience Survey Methodological Report. 2019. https://www.qilt.edu.au/docs/default-source/resources/ses/2018/2018-ses-methodological-report.pdf?sfvrsn=1f5a55a7_1

[24] Cer D, Yang Y, Kong S, Hua N, Limtiaco N, John RS. Universal sentence encoder. arXiv preprint 2018. https://arxiv.org/abs/1803.11175

[25] Hutto CJ, Gilbert EE. VADER: a parsimonious rule-based model for sentiment analysis of social media text. 2014.

[26] Agresti A. Analysis of ordinal categorical data. New Jersey: Wiley; 2001.

[27] Carvalho CM, Polson NG, Scott JG. The horseshoe estimator for sparse signals. Biometrika. 2010;97(2):465–80.

[28] Park T, Casella G. The Bayesian LASSO. J Am Statist Assoc. 2008;103(482):681–6.

[29] Knight K, Fu W. Asymptotics for lasso-type estimators. Annals Statist. 2000;28(5):1356–78.

[30] Polson NG, Scott JG, Windle J. The Bayesian Bridge. J Roy Statist Soc: Ser B (Statist Methodol). 2014;76(4):713–33.

[31] Ke X, Fan Y. Bayesian L 1/2 regression. J Comput Graph Statist. 2024;34(1):199–210. doi: 10.1080/10618600.2024.2374579

[32] Van Dyk DA, Park T. Partially collapsed Gibbs samplers: theory and methods. J Am Statist Assoc. 2008;103(482):790–6.

[33] Scott SL, Blocker AW, Bonassi FV, Chipman HA, George EI, McCulloch RE. Bayes and big data: the consensus Monte Carlo algorithm. Int J Manag Sci Eng Manag. 2016;11:78–88.

[34] Smith D, Betbeder-Matibet L. Katana. 2010. doi: 10.26190/669x-a286

[35] Gelman A. Scaling regression inputs by dividing by two standard deviations. Stat Med. 2008;27(15):2865–73. doi: 10.1002/sim.3107 17960576

[36] Bettinger EP, Long BT. Do faculty serve as role models? The impact of instructor gender on female students. Am Econ Assoc. 2005;95(2):152–7.

[37] Boring A, Ottoboni K, Stark PB. Student evaluations of teaching (mostly) do not measure teaching effectiveness. ScienceOpen Res. 2016. doi: 10.14293/s2199-1006.1.sor-edu.aetbzc.v1

