# Supplementary material for: Relationships between numerical score and free text comments in student evaluations of teaching: A sentiment topic analysis reveals the influence of gender and culture
Source: PLoS One. 2025 Jun 13;20(6):e0324619. doi: 10.1371/journal.pone.0324619 (PMC12165411; doi:10.1371/journal.pone.0324619)
Supplement: S1 File — (PDF) [file Pone.0324619.s001.pdf]

Supporting Information

1. A PCG sampler for Bayesian ordinal regression

The partially collapsed Gibbs sampler (1) speeds up convergence of Markov chain Monte Carlo by reducing the conditioning in some or all of the component draws of its parent Gibbs sampler, and hence is capable of handling the high dimensional problem considered here. We extend the Gaussian linear regression model of (2) to the Bayesian ordinal model with random effects.

To derive the PCG sampler, we first require appropriate choices of prior decomposition. Here, we adopt the following decomposition for the $L_{\frac{1}{2}}$ prior on the fixed effects in Equation (3), as follows:

$$\boldsymbol{\beta}|\tau_1^2,\ldots,\tau_p^2 \sim N_p\left(\mathbf{0},\frac{1}{\lambda^4}\boldsymbol{D}_{\tau^2}\right),\quad \boldsymbol{D}_{\tau^2}=\mathrm{diag}\left(\tau_1^2,\ldots,\tau_p^2\right)$$

$$\tau_1^2|v_1^2,\ldots,\tau_p^2|v_p^2 \sim \prod_{k=1}^{p}\mathrm{Exp}(\frac{1}{2v_k^2}),\quad v_1,\ldots,v_p \sim \prod_{k=1}^{p}\mathrm{Gamma}(\frac{3}{2},\frac{1}{4})$$

where $\boldsymbol{D}_{\tau^2}$ is a diagonal matrix, $\tau_1^2,\ldots,\tau_p^2,\nu_1^2,\ldots,\nu_p^2$ are latent parameters introduced to facilitate the decomposition. This normal-exponential-gamma mixture representation allows us to obtain simple, full conditionals that are easy to sample from using the Gibbs sampler.

(3) showed that the logistic distribution (Equation 1) can be well approximated by a t-distribution, using degree of freedom $\nu = 6.4$ and scale $\eta = 1.539$ to approximate the standard logistic distribution resulted in an error of 0.0006 for the CDF and 0.0007 for the PDF under the L_∞ norm. Using the t-distribution as an approximation and the normal-gamma mixture representation of the t, we can now express the latent variable z_i as

$$z_i|\boldsymbol{\beta},\boldsymbol{b},w_i \sim N(x_i\boldsymbol{\beta}+T\boldsymbol{b},w_i^{-1}),\quad w_i \sim \mathrm{Gamma}(\frac{\nu}{2},\frac{\nu\eta^2}{2})$$

which is then used in deriving the condition posterior for $\pi(\boldsymbol{\beta}|\boldsymbol{b},\boldsymbol{\tau}^2,\boldsymbol{Z})$. Figure 1 graphically illustrates the Bayesian ordinal model and the dependence structure of all the parameters and latent variables.

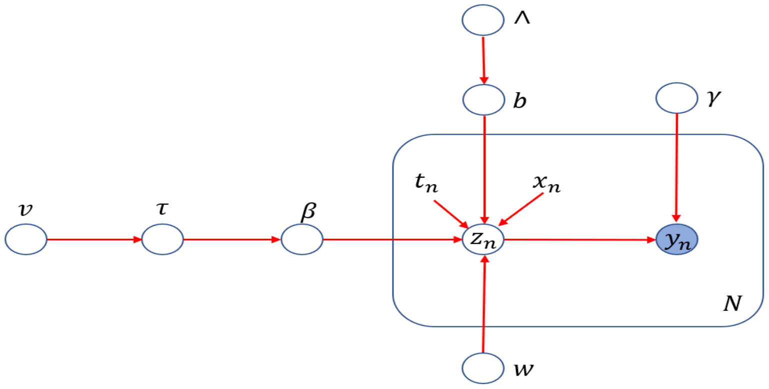

Fig 1. A graphical representation of the Bayesian ordinal regression model. The box labelled N represents N nodes of which only a single variable of t_n,x_n,z_n and y_n are shown explicitly. Shaded circle correspond to the response variable y_n.

The conditional distributions used in the PCG sampler is given below:

$$\boldsymbol{\beta}|\boldsymbol{b},\boldsymbol{W},\boldsymbol{\tau}^2,\boldsymbol{Z},\lambda \sim \mathrm{N_p}((\boldsymbol{X}^T\boldsymbol{W}\boldsymbol{X}+\lambda^4\boldsymbol{D}_{\boldsymbol{\tau}^2}^{-1})^{-1}\boldsymbol{X}^T\boldsymbol{W}(\boldsymbol{Z}-\boldsymbol{T}\boldsymbol{b}),(\boldsymbol{X}^{\boldsymbol{T}}\boldsymbol{W}\boldsymbol{X}+\lambda^4\boldsymbol{D}_{\boldsymbol{\tau}^2}^{-1})^{-1})$$
$$\boldsymbol{b}|\boldsymbol{Z},\boldsymbol{\beta},\boldsymbol{W},\boldsymbol{\Lambda} \sim N_q((\boldsymbol{T}^T\boldsymbol{W}\boldsymbol{T}+\boldsymbol{\Lambda})^{-1}\boldsymbol{T}^T\boldsymbol{W}(\boldsymbol{Z}-\boldsymbol{X}\boldsymbol{\beta}),(\boldsymbol{T}^T\boldsymbol{W}\boldsymbol{T}+\boldsymbol{\Lambda})^{-1})$$
$$\boldsymbol{\Lambda}|\boldsymbol{b} \sim \mathrm{Wishart}(\delta+1,(\boldsymbol{P}+\boldsymbol{b}\boldsymbol{b}^T)^{-1})$$
$$h_k=\frac{1}{v_k}|\beta_k,\lambda \sim \mathrm{InverseGaussian}(\sqrt{\frac{1}{4\lambda^2|\beta_k|}},\frac{1}{2}), \quad k=1,...,p$$
$$r_k=\frac{1}{\tau_k^2}|\beta_k,v_k,\lambda \sim \mathrm{InverseGaussian}(\frac{1}{\lambda^2 v_k|\beta_k|},\frac{1}{v_k^2}), \quad k=1,...,p$$
$$z_i|y_i,\boldsymbol{\beta},\boldsymbol{b},\boldsymbol{\gamma} \sim \mathrm{Logistic}(x_i\boldsymbol{\beta}+T_i\boldsymbol{b},1)1_{\gamma_{s-1}<z_i<\gamma_s}, \text{ if } y_i=s, \text{ for } i=1,...,n$$
$$w_i|z_i,\boldsymbol{\beta},\boldsymbol{b} \sim \mathrm{Gamma}(\frac{\nu}{2},\frac{\nu\eta^2+(z_i-x_i\boldsymbol{\beta}-T_i\boldsymbol{b})^2}{2}), \quad i=1,...,n$$
$$\gamma_s|\gamma_{-s},\boldsymbol{Y},\boldsymbol{Z} \propto f(\gamma_s)1_{l_s<\gamma_s<u_s}$$

2. The PCG algorithm

Here we give the three steps that comprise the PCG sampler.
Step M: Marginalise

Step 1: Draw $\boldsymbol{\beta}$ from $\pi(\boldsymbol{\beta}|\boldsymbol{b},\boldsymbol{W},\boldsymbol{\tau}^2,\boldsymbol{Z},\lambda)$
Step 2: Draw $\boldsymbol{\tau}^2$ from $\pi(\boldsymbol{\tau}^2|\boldsymbol{\beta},\boldsymbol{v},\lambda)$
Step 3: Draw $\boldsymbol{b}$ from $\pi(\boldsymbol{b}|\boldsymbol{Z},\boldsymbol{\beta},\boldsymbol{W},\Lambda)$
Step 4: Draw $\boldsymbol{\Lambda}$ from $\pi(\boldsymbol{\Lambda}|\boldsymbol{b})$
Step 5: Draw ${\boldsymbol{\tau}^2}^\star$ and $\boldsymbol{v}$ from $\pi(\boldsymbol{\tau}^2,\boldsymbol{v}|\boldsymbol{\beta},\lambda,\boldsymbol{b},\boldsymbol{Y})$
Step 6: Draw z_i and $w_i^\star$ from $\pi(z_i,w_i|\boldsymbol{\beta},\boldsymbol{b},\gamma,\boldsymbol{Y}), \quad i=1,\ldots,n$
Step 7: Draw w_i from $\pi(w_i|z_i,\boldsymbol{\beta},\boldsymbol{b}), \quad i=1,\ldots,n$
Step 8: Draw γ_s from $\pi(\gamma_s|\gamma_{s-1},\gamma_{s+1},\boldsymbol{Z},\boldsymbol{Y}), \;\; s=1,\ldots,S-1$

Step P: Permute

Step 1: Draw $\boldsymbol{\beta}$ from $\pi(\boldsymbol{\beta}|\boldsymbol{b},\boldsymbol{W},\boldsymbol{\tau}^2,\boldsymbol{Z},\lambda)$
Step 2: Draw $\boldsymbol{b}$ from $\pi(\boldsymbol{b}|\boldsymbol{Z},\boldsymbol{\beta},\boldsymbol{W},\boldsymbol{\Lambda})$
Step 3: Draw $\boldsymbol{\Lambda}$ from $\pi(\boldsymbol{\Lambda}|\boldsymbol{b})$
Step 4: Draw ${\boldsymbol{\tau}^2}^\star$ and $\boldsymbol{v}$ from $\pi(\boldsymbol{\tau}^2,\boldsymbol{v}|\boldsymbol{\beta},\lambda,\boldsymbol{b},\boldsymbol{Y})$
Step 5: Draw $\boldsymbol{\tau}^2$ from $\pi(\boldsymbol{\tau}^2|\boldsymbol{\beta},\boldsymbol{v},\lambda,\boldsymbol{b},\boldsymbol{Y})$
Step 6: Draw z_i and $w_i^\star$ from $\pi(z_i,w_i|\boldsymbol{\beta},\boldsymbol{b},\gamma,\boldsymbol{Y}), \quad i=1,\ldots,n$
Step 7: Draw w_i from $\pi(w_i|z_i,\boldsymbol{\beta},\boldsymbol{b}), \quad i=1,\ldots,n$
Step 8: Draw γ_s from $\pi(\gamma_s|\gamma_{s-1},\gamma_{s+1},\boldsymbol{Z},\boldsymbol{Y}), \;\; s=1,\ldots,S-1$

Step T: Trim

Step 1: Draw $\boldsymbol{\beta}$ from $\pi(\boldsymbol{\beta}|\boldsymbol{b}, \boldsymbol{W}, \boldsymbol{\tau}^2, \boldsymbol{Z}, \lambda)$
Step 2: Draw $\boldsymbol{b}$ from $\pi(\boldsymbol{b}|\boldsymbol{Z}, \boldsymbol{\beta}, \boldsymbol{W}, \boldsymbol{\Lambda})$
Step 3: Draw $\boldsymbol{\Lambda}$ from $\pi(\boldsymbol{\Lambda}|\boldsymbol{b})$
Step 4: Draw $\boldsymbol{v}$ from $\pi(\boldsymbol{v}|\boldsymbol{\beta}, \lambda)$
Step 5: Draw $\boldsymbol{\tau}^2$ from $\pi(\boldsymbol{\tau}^2|\boldsymbol{\beta}, \boldsymbol{v}, \lambda)$
Step 6: Draw z_i from $\pi(z_i|\boldsymbol{\beta}, \boldsymbol{b}, \boldsymbol{\gamma}, \boldsymbol{Y}), \quad i = 1, \ldots, n$
Step 7: Draw w_i from $\pi(w_i|z_i, \boldsymbol{\beta}, \boldsymbol{b}), \quad i = 1, \ldots, n$
Step 8: Draw γ_s from $\pi(\gamma_s|\gamma_{s-1}, \gamma_{s+1}, \boldsymbol{Z}, \boldsymbol{Y}), \quad s = 1, \ldots, S-1$

We use superscript $\star$ to present intermediate quantities that are sampled but not retained as part of the output. Step M is a generalisation of the traditional Gibbs sampler with some components being updated multiple times within each iteration. In the permute step we rearranged the ordering of the update in Step M. Finally, in the Trim step, the intermediate draws of $\boldsymbol{\tau}^{2\star}$, $\boldsymbol{v}^\star$ and $w^\star$ are not necessary if we can sample $\boldsymbol{v}$ and $\boldsymbol{Z}$ in step 4 and step 6 directly from the respective marginal distributions.

3. Model estimates

VARIABLE	ART	COM	ENG	MED	SCI	CMC
Postgraduate Course	0.03	**0.14**	**0.20**	0.02	**0.39**	**0.13**
Research Course	**0.64**	**1.16**	**1.19**	0.10	**0.49**	**0.70**
Lecturer Background Unknown	**-0.32**	**-0.19**	**-0.26**	-0.15	**0.22**	**-0.12**
Lecturer Non-English Speaking (LNE)	0.15	**-0.35**	**-0.14**	-0.16	0.00	**-0.07**
Student Male (SM)	0.05	0.02	-0.02	0.06	0.01	0.02
Student Local (SL)	**0.10**	-0.05	**-0.19**	0.15	0.05	-0.02
Total Students	**-0.40**	**-0.08**	**-0.77**	**-0.30**	**0.06**	**-0.06**
Lecturer Female (LF)	**0.18**	**-0.16**	-0.02	0.06	-0.08	0.00
Semester WAM	**0.11**	**0.13**	**0.24**	**0.23**	**0.18**	**0.12**
Staff Quality Sentiment (SQ)	**0.22**	**0.36**	**0.39**	**0.27**	**0.29**	**0.24**
Teaching Method Sentiment (TM)	**0.19**	**0.15**	**0.13**	0.07	**0.23**	**0.12**
Miscellaneous Sentiment (MS)	**0.11**	**0.33**	**0.22**	0.01	**0.16**	**0.16**
SL x LNE	-0.14	**-0.23**	-0.15	0.00	0.05	**-0.09**
SL x LF	0.04	**-0.14**	-0.01	-0.12	**0.17**	0.04
LF x LNE	-0.14	**0.40**	-0.06	-0.11	-0.11	0.03
SL x LF x LNE	-0.26	0.02	-0.08	-0.33	**-0.73**	**-0.13**
SM x SL x LF	0.01	0.02	0.00	-0.05	**0.59**	0.04
MS x SM	0.02	0.03	0.10	0.04	0.09	**0.05**
MS x SL	-0.03	**-0.14**	-0.04	-0.03	**-0.14**	-0.08
SQ x LNE	0.02	**0.13**	0.00	0.05	-0.03	0.02

Table 1. Estimates of regression coefficients using SET score as response. An 'x' represents an interaction between the variables. Results in **bold** indicate significance at the 95% confidence interval. Columns correspond to the faculties of Art, Commerce, Engineering, Medicine, Science and aggregate results on the whole university (CMC). Coefficients with no significant effects are omitted from the table.

VARIABLE	ART	COM	ENG	MED	SCI	CMC
Postgraduate Course	**-0.39**	**-0.53**	**-0.21**	-0.16	**-0.53**	**-0.38**
Research Course	**0.78**	**0.73**	**0.68**	0.14	**0.58**	**0.70**
Undergraduate Course	**-0.45**	**-0.22**	-0.14	-0.04	**-0.43**	**-0.29**
Lecturer Background Unknown	**-0.15**	**-0.27**	**-0.11**	-0.09	**-0.08**	**-0.14**
Lecturer Non-English Speaking LNE	0.04	**-0.25**	**-0.17**	-0.15	**-0.25**	**-0.16**
Student Local (SL)	**0.48**	**0.53**	**0.42**	0.00	**0.47**	**0.43**
Lecturer Female (LF)	**0.20**	0.10	0.07	0.19	0.04	**0.13**
Total Students	**-0.33**	**-0.36**	**-0.71**	-0.01	**-0.24**	**-0.28**
Semester WAM	**0.42**	**0.36**	**0.25**	**0.22**	**0.27**	**0.32**

Table 2. Estimates of regression coefficients using sentiment of SQ as response. Results in **bold** indicate significance at the 95% confidence interval. Columns correspond to the faculties of Art, Commerce, Engineering, Medicine, Science and aggregate results on the whole university (CMC). Coefficients with no significant effects are omitted from the table.

VARIABLE	ART	COM	ENG	MED	SCI	CMC
Postgraduate Course	**-0.40**	**-0.56**	**-0.49**	-0.08	**-0.66**	**-0.36**
Lecturer Background Unknown	**-0.12**	-0.05	**-0.20**	-0.27	-0.02	**-0.13**
Lecturer Non-English Speaking LNE	-0.01	**-0.20**	-0.09	0.01	-0.13	**-0.14**
Student Male (SM)	**-0.19**	-0.12	-0.07	-0.19	**-0.20**	**-0.05**
Student Local (SL)	**0.38**	**0.79**	**0.74**	0.06	**0.59**	**0.46**
Lecturer Female (LF)	0.04	0.00	0.04	0.01	0.04	**0.14**
Total Students	-0.06	**-0.23**	**-0.24**	0.11	**-0.19**	**-0.26**
Semester WAM	**0.49**	**0.34**	**0.25**	0.14	**0.27**	**0.33**
SM x SL	-0.01	**-0.18**	-0.15	0.08	0.03	0.00

Table 3. Estimates of regression coefficients, using sentiments of TM as response. An 'x' represents an interaction between the variables. Results in **bold** indicate significance at the 95% confidence interval. Columns correspond to the faculties of Art, Commerce, Engineering, Medicine, Science and aggregate results on the whole university (CMC). Coefficients with no significant effects are ommited from the table.

VARIABLE	ART	COM	ENG	MED	SCI	CMC
Postgraduate Course	**0.21**	0.03	**0.22**	0.11	0.05	**0.14**
Lecturer Background Unknown	-0.02	0.00	-0.06	-0.23	**-0.35**	**-0.07**
Lecturer Non-English Speaking LNE	-0.03	**-0.20**	-0.07	0.08	-0.14	**-0.09**
Student Male (SM)	0.02	0.03	-0.01	0.06	0.11	**0.06**
Student Local (SL)	**-0.25**	**-0.24**	**-0.37**	-0.09	**-0.27**	**-0.27**
Lecturer Female (LF)	-0.01	-0.12	-0.08	0.12	**-0.20**	**-0.08**
Total Students	**0.14**	**-0.15**	**-0.19**	**-0.58**	-0.02	**-0.05**
Semester WAM	**-0.22**	**-0.18**	-0.06	-0.07	**-0.15**	**-0.15**

Table 4. Estimates of regression coefficients, using sentiments of MS as response. Results in **bold** indicate significance at the 95% confidence interval. Columns correspond to the faculties of Art, Commerce, Engineering, Medicine, Science and aggregate results on the whole university (CMC). Coefficients with no significant effects are ommited from the table.

References

1. Van Dyk DA, Park T. Partially collapsed Gibbs samplers: Theory and methods. Journal of the American Statistical Association. 2008;103(482):790–796.

2. Ke X, Fan Y. Bayesian $L_{\frac{1}{2}}$ regression. Journal of Computational and Graphical Statistics. 2024;p. 1–12.

3. Pingel R. Some approximations of the logistic distribution with application to the covariance matrix of logistic regression. Statistics and Probability Letters. 2014;85(63-68).
